# Individual Variation in the Use of Acoustic Signals to Coordinate Group Movements among Tibetan Macaques (*Macaca thibetana*)

**DOI:** 10.3390/ani12162149

**Published:** 2022-08-22

**Authors:** Meng-Meng Chen, Yu-Heng Zhang, Yi-Mei Tai, Xi Wang

**Affiliations:** 1 School of Resources and Environmental Engineering, Anhui University, Hefei 230601, China; 2 International Collaborative Research Center for Huangshan Biodiversity and Tibetan Macaque Behavioral Ecology, Hefei 230601, China

**Keywords:** Tibetan macaques, collective movements, vocal communication, group coordination, decision making, social networks

## Abstract

**Simple Summary:**

Vocal communication is widely used in most primate groups as one of the most effective ways to transmit information. However, the role of sound signals in group movements and their influencing factors are not well understood. In this study, we not only confirmed the recruitment function of vocalizations in group movements, but also found the effect of sex and social centrality on vocalizations. Social centrality indicates the degree of proximity relations between two individuals in a social network. Female Tibetan macaques and individuals with high social centrality were more likely to use vocalizations during collective movements. This study helps us understand the cooperative mechanisms of animal populations.

**Abstract:**

To maintain group cohesion, social animals need to coordinate their actions during group movements. Several species use vocalizations to communicate with each other during coordination. However, the process of vocal communication and its influence in collective decision making is not clear. We studied a group of free-range Tibetan macaques (*Macaca thibetana*) at Huangshan, China, and recorded acoustic signals during their group movements. It was found that three kinds of sounds were used in their movements. Group movements with vocalizations recruited more participants than the movements without sound. Moreover, during group departures, individuals in the front emitted a higher frequency of vocalization than individuals in the rear. Sex and social centrality both had a significant influence on vocalizations. Social centrality indicates the degree of proximity relations between two individuals in a social network. Females and individuals with high social centrality emitted more sound in group movements. However, social rank and the number of relatives did not affect the emission of sound. These results suggest that the function of calls in collective movements relates to coordinating group movements. This study provides an insight into the association of acoustic communication with collective decision making.

## 1. Introduction

Group living is beneficial to the cooperation of animals for foraging, defense, energy conservation and reproduction [1,2]. However, considering that the interests and needs of different members of a group are not necessarily the same, group cohesion may be weakened, and the advantages associated with collective actions may be decreased [3]. The basis for the success of animals living in groups is that each individual must cooperate to ensure the unity of the group. Otherwise, the advantages of being in the group cease to exist, with each member facing the challenge of survival.

In some animal groups, individuals are only aware of the situation of the neighboring members, and it is difficult for information to be effectively and quickly transmitted through the group [4]. However, being familiar with neighboring individuals and the information of the whole group makes the decision-making process of the animals more complex and effective [5]. During collective decision making, the initiators communicate their preferences on movement direction and time to other members by the mode of behavioral interaction and information exchange with neighboring members [6]. The correct use of communication behavior is crucial to successful collective decision making as well as the achievement of group movements [7].

Acoustic signals can overcome the obstruction of sight, have the characteristics of strong real-time performance and high transmission efficiency, and help to realize information exchange between individuals or groups [8]. Accordingly, several species use calls to maintain relations between group members during group movements [9]. For example, before moving to the open grasslands from dense forest, meerkats (*Suricata Suricatta*) inform peers of their willingness to leave through auditory communication [10]. African wild dogs (*Lycaon pictus*) use acoustic signals to initiate group movements [11]. As well as that, apostlebirds (*Struthidea cinerea*) emit calls to coordinate the actions of members before the group departs [12]. Vocal communication can thus maintain group stability [13].

In primates, vocal signals exert significant impacts on group movements. Calling can send information about a propensity to take certain actions, which can facilitate the probability of collective movements [14]. Studies have shown that vocalization may serve as a means of initiating collective movements. For example, red-fronted lemurs (*Eulemur rufifrons*) use close calls (grunts) as an announcement before group departures to coordinate collective action [15]. Similar evidence has also been reported in other primates, such as white-faced capuchin monkeys (*Cebus capucinus*) [16], barbary macaques (*Macaca sylvanus*) [17], and Tonkean macaques (*M. tonkeana*), where intragroup vocal signals are used for initiating group movements [18].

Vocal signals can be used in group recruitments before departure or at the beginning of movements [15]. In a group of wild bonobos (*Pan paniscus*), group leaders employ specific call combinations, i.e., the “low hoot–high hoot”, to recruit other individuals for group travel [19]. Similarly, a pilot study on a Namibian population of baboons (*Papio ursinus*) indicated that the likelihood of members following increases with the number of grunts by individuals [5]. Primates have high cognitive ability and complex social relationships. Individuals not only have local cognition of the behaviors of neighbors, but also are aware of the overall information of the environment. The function of vocal communication, therefore, becomes more diverse and complex [20]. It is necessary to further explore the types and processes of acoustic signals combined with different social factors.

Studies on Tibetan macaques (*M. thibetana*) showed that visual communication was used to recruit and monitor group members during group movements [21]. However, there is a lack of research on vocal communication among Tibetan macaques. Furthermore, it is necessary to explore the process of vocalizations in primates. Thus far, it has been confirmed that there is frequent vocal communication during group movements in Tibetan macaques [20]. However, the functions and types of sound in the decision-making processes are not clear.

Preliminary observations indicate that vocal communication often occurs before and during group movements of Tibetan macaques. To analyze the process of vocal communication and its influence on the collective decision making in macaques, it was predicted that: (1) calls would play a role in recruiting movement participants, and if so, individuals in the front position in the group movement would be more likely to emit vocalization; (2) social factors would affect the frequency of vocalization, and, if so, that females or individuals with a higher rank, higher social centrality or with a larger number of relatives would vocalize more frequently during collective movements. Because this species lives in a matrilineal society, the social networks of females are essential for maintaining group stability.

## 2. Materials and Methods

### 2.1. Study Site and Subjects

The current study was performed in Mt. Huangshan, China, at the field site in the Valley of the Wild Monkeys. Yulinkeng 1 group (YA1) was selected as the study group.

The study was conducted from 18 August 2021 to 18 January 2022. The study group comprised 49 monkeys (10 adult males, 12 adult females, 2 sub-adult males, 3 sub-adult females, 15 juveniles and 7 infants) during the observation. All adults and sub-adults were considered as focal animals (Table 1). Individuals in this group have been studied continuously since 1986. Thus, all group members have been accustomed to the presence of observers and they have been individually identified based on unique physical features, such as scars, hair color and facial appearance [22]. In this study, the research group spent most of the day in the forest. Regularly, the monkeys were provided with a small amount of corn in an open area with high visibility every day [23]. After corn foraging, the monkeys would stay in the provisioning area for a while and then return to the forest.

### 2.2. Data Collection

#### 2.2.1. Behavioral Observations and Definitions

The behavioral data were observed and collected by one observer. Daily observation for the study group lasted 6.5 h from 08:30–11:30 and 14:00–17:30. Group movements were collected by all-occurrence recording methods [25], and the aggression–submission bouts were recorded with the ad libitum sampling method using a video camera (SONY China, Beijing, China). Focal animals (N = 27) were observed using continuous sampling [25] during the 10-min observation sessions using an audio recorder (Lenovo China, Beijing, China).

Aggressive acts were displayed as staring, chases, slaps, grabs or bites, while submissive acts were displayed as bared teeth, mock leave, avoidance, fleeing or screaming [26]. Table 2 presents the definition of the behaviors in detail [22,27,28,29,30,31].

#### 2.2.2. Vocalization Recording and Acoustic Analyses

During group movements, the identity of the caller was recorded using an audio recorder. Continuous sampling was used to score the vocalizations in the context of group movements. The vocalizations were documented with the Tascam DR44-WL (Tascam, Japan) digital recorder with a sampling rate of 44.1 kHz (16 bits) in connection with the Sennheiser MKE600 (Sennheiser, Germany) directional microphone.

The vocalization data were stored on a laptop computer for further analysis. Spectrograms of the audio files were created with the Praat 6.2.10 package (Gaussian window shape, view range = 0–5 kHz, window length = 0.03 s, dynamic range = 100 Db: Boersma and Weenink, University of Amsterdam, the Netherlands). Table 3 presents definitions for the acoustic parameters used in vocalization [32].

### 2.3. Data Analysis

The order of individuals was arranged in every collective movement. During group departures, the time of departure of each individual joining a movement was scored. The order of adults with the formula 1−[I−1/N−1] was then measured, where I indicates the position where the individual joins the movement, and N represents the number of participants involved in the movement [33]. The index ranged between 1 (first position) and 0 (last position). The higher the index, the earlier the individual joins the movement.

The study used the dyadic association index (DAI) to measure the affiliative interaction of individuals A and B. DAI = D_ab_/(D_a_ + D_b_ − D_ab_), where D_ab_ indicates the total duration of 1 m proximity between individual A and individual B, D_a_ and D_b_, respectively, refer to the total focal sampling duration of individuals A and B [34]. Based on the DAI matrix, the eigenvector centrality coefficient for every individual was measured using UCINET v6.2. NETDRAW v2.0 was then applied to draw the social network.

David’s Score (DS) was measured to determine the dominance rank of the adults [24]. Dyadic agonistic (aggressive/submissive) interactions were referenced for dominance [22,35]. A larger DS value corresponds to a higher social rank.

Two individuals belonging to the same matrilineal line (i.e., relatives, Table 1) were considered to be related regardless of their degree of relatedness [21,36]. The kinship coefficient was amended as the number of individuals in a group connected through matrilineal kinship [21]. The number of migrant relatives for this group was considered as 0.

### 2.4. Statistical Analysis

Based on the observations, we found that individuals were not using vocalization in every single movement at all times, so there were several zero values that corresponded to sound signals. To solve this issue of multiple zeros for dependent variables, a new R package, glmmTMB [37], was adopted by constructing generalized linear mixed models (GLMM) to test whether sex, social centrality, social rank and relatives affected the vocalization frequency during collective movements. Mann–Whitney U test was used to examine the difference in the number of participants in the group movements with or without sound communication. The correlation between the joining order and vocalization rates was analyzed using Spearman’s rank correlation test.

All analyses were conducted using R version 4.1.2. The significance level was 0.05. All tests were two-tailed, with the mean (±SE).

### 2.5. Ethics Statement

This study was conducted with the Huangshan Monkey Management Center and the Huangshan Garden Forest Bureau. It complies with the regulations of the Chinese Wildlife Conservation Association regarding the ethical treatment of research subjects, and under the law of People’s Republic of China on the protection of wildlife. The study was completely observational in nature and did not affect the welfare of the monkeys.

## 3. Results

### 3.1. The Types of Sound Signals during Group Movements

**Figure 1:**
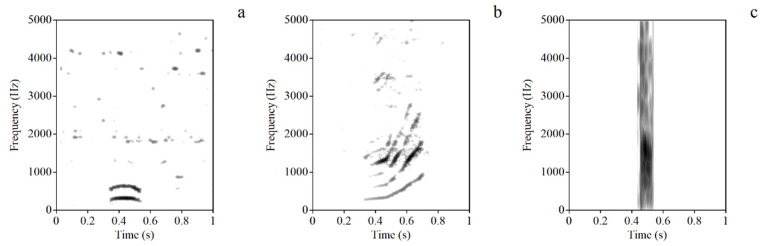
Representative spectrogram vocalizations. (**a**) *Coo*; (**b**) *leap coo*; (**c**) *bark*.

In this study, a total of 30 high-quality sounds were collected, which could be visually distinguished in spectrograms (Figure 1). According to the sound parameters obtained from the analysis, the vocalizations (Table 4) were categorized as coo (N = 22), leap coo (N = 7) and bark (N = 1).

### 3.2. The Usage of Vocal Communication during Group Movements

During the study period, a total of 132 successful group movements were recorded, of which 52 demonstrated vocal communication, accounting for 39.4%, and 80 demonstrated no vocal communication, accounting for 60.6%. Among the 52 group movements with vocal communication, the initiators demonstrated vocal communication 34 times, accounting for 65.3%.

### 3.3. The Number of Participants during Group Movements

**Figure 2:**
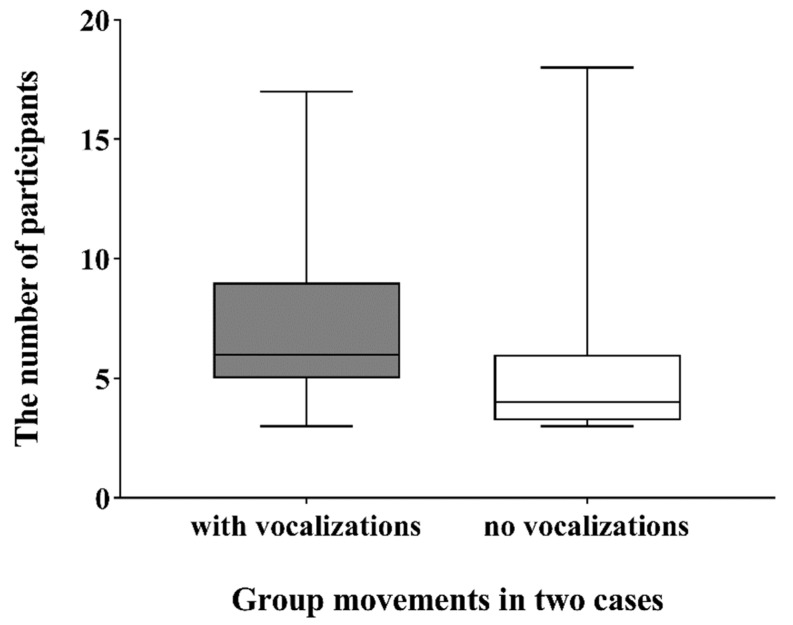
The number of participants in group movements with or without vocal communication.

To test the effect of vocal communication on the number of participants in group movements, we analyzed the difference on the number of participants when there were vocalizations and when there were none. The result showed that vocalizations have a significant impact on the number of participants (Mann–Whitney U test: W = 2845.5, *p* < 0.001). Group movements with vocalizations recruited more participants than the movements without vocalizations (Figure 2).

### 3.4. Joining Order and Vocalization

**Figure 3:**
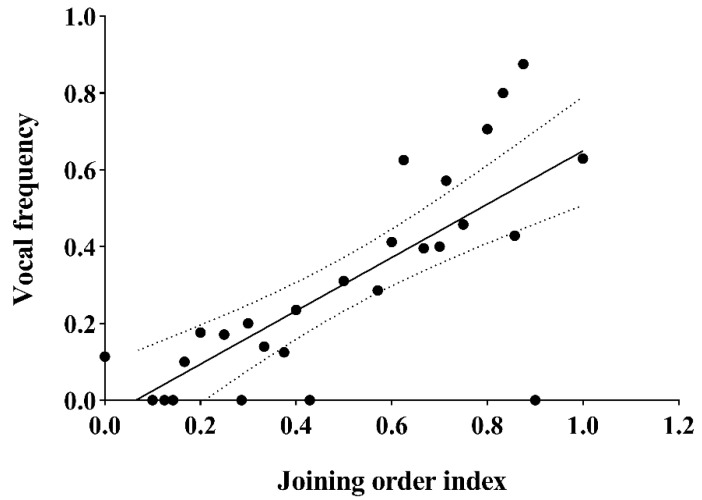
The correlation between the joining order index and vocal frequency during collective movements. The horizontal axis represents the joining orders of individuals: the higher the index, the earlier the individual joins the movement. The vertical axis represents the vocal frequency of individuals. The larger the vocal frequency, the more frequently the individual vocalizes.

To analyze the relationship between an individual’s position in the group movement and its vocalization, a correlation analysis was performed between the order index and vocalization frequency. The results demonstrated that there was a positive correlation between the order index and individual vocal frequency (Spearman’s rank correlation: N = 27, r = 0.758, *p* < 0.001, Figure 3).

### 3.5. Social Factors and Vocalization

**Figure 4:**
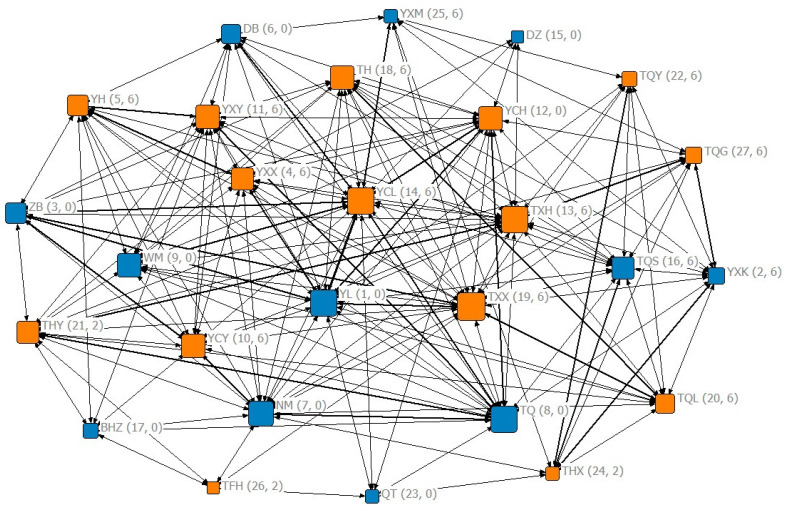
Eigenvector centrality coefficients for group members within the social network. Nodes indicate individuals; females and males are marked in orange and blue, respectively; the labels indicate the name of an individual; the size of the node indicates the value of eigenvector centrality; the thickness of links indicates the degree of proximity relations; the pair of numbers in the bracket represents social rank and number of relatives, respectively.

**Figure 5:**
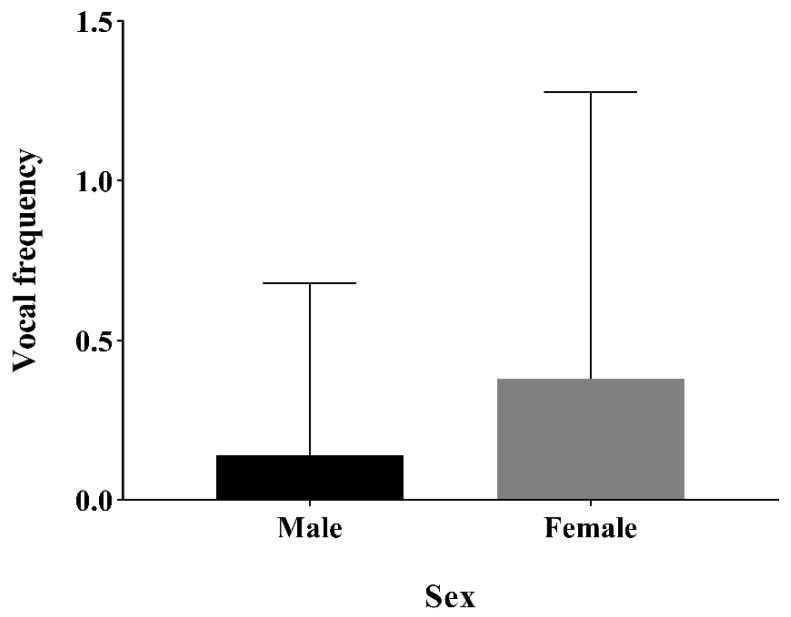
Differences in vocal frequency between males and females during collective movements. The vertical axis represents the vocal frequency of individuals. The larger the vocal frequency, the more frequently the individual vocalizes.

A social network was drawn, which comprised sex, social centrality and social rank as well as the number of relatives (Figure 4). GLMM was then used to analyze the influence of each variable on the frequency of calls. The results demonstrated that sex had a significant effect on the vocal frequency (*p* < 0.01), namely, the females called more often than males when initiating or following group movements (Table 5, Figure 5). Social centrality also significantly affected the vocal frequency (*p* < 0.05, Table 5). However, social rank (*p* = 0.619, Table 5) and the number of relatives (*p* = 0.978, Table 5) had no significant effect on the vocal frequency.

## 4. Discussion

Vocal communication is widely used in most primate groups as one of the most effective ways to transmit information. For example, Chacma baboons (*Papio ursinus*) utter grunts to initiate a group movements [38], while wild chimpanzees (*Pan troglodytes*) use “travel hoo” for joint travel [39]. The current study explored the use of vocalization in coordinating collective movements among Tibetan macaques. The results showed that about 39.4% of collective movements used vocal communication, while 60.6% had no vocalizations. This may be an energy-saving strategy since, in most cases, they do not need to make a vocalization to promote the success of the collective movements. It was found that three types of sounds were used in the collective movements of macaques. We also found that the joining order was positively correlated with the vocal frequency of individuals in collective movements. These results are similar to the study on white-faced capuchins [16]. Furthermore, sex and social centrality were found to be the primary influencing factors of vocalizations among Tibetan macaques’ group movements.

### 4.1. Recruitment Function of Acoustic Signals

In Tibetan macaques, group movements occurred frequently, but not all group movements had vocalizations. We compared the number of participants with vs. without vocal communication, and found that the number of participants in the movements with vocalizations was significantly greater than that without vocalizations. Moreover, in the group movements with sound communication, the earlier the individuals joined movements, the greater the frequency of calls they emitted. We suggest that the callers used sound signals to convey information about their spatial location while influencing the behavior of other individuals, thereby indicating that the calls may act as a signal to notify or attract other individuals to move with them. Acoustic communication maintains or reduces the distance between group mates, increasing the likelihood that most members follow the initiators [5]. This has been confirmed in several species, such as bonobos [19], red-fronted lemurs [15] and white-faced capuchins [16]. The results of this study may indicate the recruitment function of sound in the collective movement of Tibetan macaques.

### 4.2. Influence of Sex and Social Factors on Frequency of Acoustic Signals

This study revealed that sex affected the vocal frequency, wherein females emitted more vocalizations than males in group movements. This may be related to the social style of Tibetan macaques. Previous studies have demonstrated that this species lives in a matrilineal society, where females live in the natal group throughout their lifetime (except for the group fission period), but males move away from the group upon sexual maturity [22]. For macaque species featuring female philopatry and male dispersal, males have weak social bonds with other resident individuals after entering the new group [40]. During the collective movements, female Tibetan macaques used vocal communication to coordinate the actions among group members to avoid group dispersion. This is similarly observed in Barbary macaques, where female individuals were reported to make “soft yells” very frequently when moving in groups [17]. Females, therefore, play an important role in maintaining social cohesion [41].

This study also demonstrated that social centrality had significant effects on the vocalization frequency during collective movements. Previous studies have shown that social centrality plays a key role in the success of collective movements in Tibetan macaques, where individuals demonstrating higher social centrality are more likely to succeed in initiating the group movement [28]. The eigenvector centrality coefficient measures how closely associated an individual is to others in group movements [42]. Thus, it is suggested that individuals with high social centrality possessed a stronger social affinity and could interact with more followers through vocal communication.

The social rank did not affect the vocal frequency, indicating that the species may fit better with distributed leadership in group movements. Tibetan macaques belong to a strictly hierarchical social system [22], but they share the decision making during the initiation of the collective movements [43]. In this study, it was found that members, regardless of rank, can initiate or participate in the group move through vocal communication. Similarly, in ring-tailed lemurs (*Lemur catta*), low-ranking male individuals “hum” during their movements [44]. In addition, our results can be explained by the mating competition. The present study was performed in the mating season (August–January). Low-ranking individuals usually stay at the edge of the group for a while when others start moving. They might, therefore, use vocalizations in search of mating opportunities.

Furthermore, we found that the number of relatives did not affect the vocal frequency in Tibetan macaques. This may be related to the group cohesion when monkeys initiated collective movements. Group movements often occurred when macaques moved from one place to another after resting. During the observation, individuals belonging to the same matrilineal line often sat together or engaged in social grooming. The distance between relatives was, therefore, very short. When group departure occurred, one of the members started to move, and his/her relatives would closely follow [45], without sound signals for communication.

Our study was conducted during the mating season, so it is unclear whether vocal communication performs the same function during the non-mating season. In addition, only the Yulinkeng 1 group were studied, and the number of vocalizations collected was small, so it is necessary to compare with other groups of Tibetan macaques to determine the role of vocal communication in collective movements. Although the present study explored the category of sound signals, it is unclear whether the receiver could precisely discriminate between these calls. Further studies can take into account the receiver’s responses by replaying experiments.

## 5. Conclusions

In conclusion, our study led to three findings. First, we found that three types of sounds were used in collective movements. Second, vocalizations exerted a significant influence on the collective movements of Tibetan macaques. Callers can coordinate group movements or recruit other individuals through sound signals, avoiding the risk of group separation and increasing group cohesion. Third, female Tibetan macaques and individuals with high social centrality were more likely to use vocalizations during collective movements. This study provides an insight into the association of acoustic communication with collective decision making, which helps us understand the cooperative mechanisms of animal populations.

## Figures and Tables

**Table 1:** Characteristics of focal animals from the YA1 group in observation.

Individual	Sex	Number of Relatives	Social Rank	David’s Score ^#^
YL	Male	0	1	205.24
YXK	Male	6	2	176.94
ZB	Male	0	3	137.17
YXX	Female	6	4	125.5
YH	Female	6	5	82.63
DB	Male	0	6	81.58
NM	Male	0	7	71.93
TQ	Male	0	8	61.68
WM	Male	0	9	39.85
YCY	Female	6	10	28.62
YXY	Female	6	11	25.08
YCH	Female	0	12	2.04
TXH	Female	6	13	−14.6
YCL	Female	6	14	−23.26
DZ	Male	0	15	−24.75
TQS	Male	6	16	−29.32
BHZ	Male	0	17	−41.48
TH	Female	6	18	−48
TXX	Female	6	19	−68.08
TQL	Female	6	20	−76.17
THY	Female	2	21	−85.9
TQY	Female	6	22	−93.89
QT	Male	0	23	−113.9
THX	Female	2	24	−138.21
YXM	Male	6	25	−145.67
TFH	Female	2	26	−150.35
TQG	Female	6	27	−153.02

^#^ David’s Score [24] was measured to determine the dominance rank of the adults. A larger DS value corresponds to a higher social rank.

**Table 2:** Behavioral definitions.

Catalog	Definition
Group movement	When no more individuals join the movement within five minutes after the joining of the last one, the number of participants, the initiator included, should be at least 3.
Initiator	The individual who first walks over 10 m within 30 s.
Follower	The individual who moves over 5 m within 45° along the direction of the initiator.
Proximity	At least two individuals maintain the sitting or lying posture within 1 m.

**Table 3:** Definitions of acoustic parameters in vocalization.

Parameters	Definitions
Duration	Duration of the entire call (s)
Mean *f*_0_	Mean frequency of the fundamental frequency contour (Hz)
Min *f*_0_	Minimum frequency of the fundamental frequency contour (Hz)
Max *f*_0_	Maximum frequency of the fundamental frequency contour (Hz)
Mean AMP	Mean intensity (amplitude) of the entire call (dB)
Min AMP	Minimum intensity (amplitude) of the entire call (dB)
Max AMP	Maximum intensity (amplitude) of the entire call (dB)

**Table 4:** Acoustic parameter summaries for each call type.

Call Type	N	Duration(s)	Mean *f*_0_	Min *f*_0_	Max *f*_0_	Mean AMP	Min AMP	Max AMP
Coo	22	0.23 ± 0.04	282 ± 29	244 ± 36	303 ± 42	53 ± 10	43 ± 10	56 ± 10
Leap coo	7	0.26 ± 0.11	361 ± 15	260 ± 60	490 ± 45	58 ± 6	45 ± 5	63 ± 6
Bark	1	0.06	378	360	398	73	68	75

**Table 5:** Social factors influencing the vocal frequency tested using GLMM.

Factors	Estimate ± SE	Z	*p*
Sex	0.771 ± 0.283	2.723	<0.01
Rank	−0.007 ± 0.013	0.498	0.619
Relatives	−0.001 ± 0.039	−0.027	0.978
Centrality	3.361 ± 1.490	2.245	<0.05

## Data Availability

The data that support the findings of this study are available from the corresponding author, upon reasonable request.
